# Supplementary material for: Quantifying the impact of uncertainty on threat management for biodiversity
Source: Nat Commun. 2019 Aug 8;10:3570. doi: 10.1038/s41467-019-11404-5 (PMC6687751; doi:10.1038/s41467-019-11404-5)
Supplement: Supplementary file 1 — Supplementary Information [file 41467_2019_11404_MOESM1_ESM.pdf]

Supplementary Information

Quantifying the impact of uncertainty on threat management for biodiversity

Nicol S, Brazill-Boast J, Gorrod E, McSorley A, Peyrard N, Chadès I.

Supplementary Figures and Tables

Supplementary Figure 1: Per-species expected gain in persistence under current uncertainty for each species group represented as percentile ranks………………………. 2

Supplementary Figure 2: Total expected gain in persistence summed over all species, under current uncertainty for each species group, as percentile ranks………….. 3

Supplementary Figure 3: Per-species expected EVPI for each species group represented as percentile ranks……………………………………………………………………………………. 4

Supplementary Figure 4: Total expected EVPI for each species group, summed over all species, represented as percentile ranks……………………………………………………. 5

Supplementary Figure 5: Schematic of the process used to provide an initial estimate of the benefit (i.e. gain in persistence) of managing a species group……………… 6

Supplementary Table 1: Number of listed species and ecological communities under State (New South Wales) and National (Australia) legislation …………………………. 7

Supplementary Table 2: Number of experts who contributed elicited data for each KTP…… 8

Supplementary Table 3: Glossary of terms used in the text…….. 10

Supplementary Figures

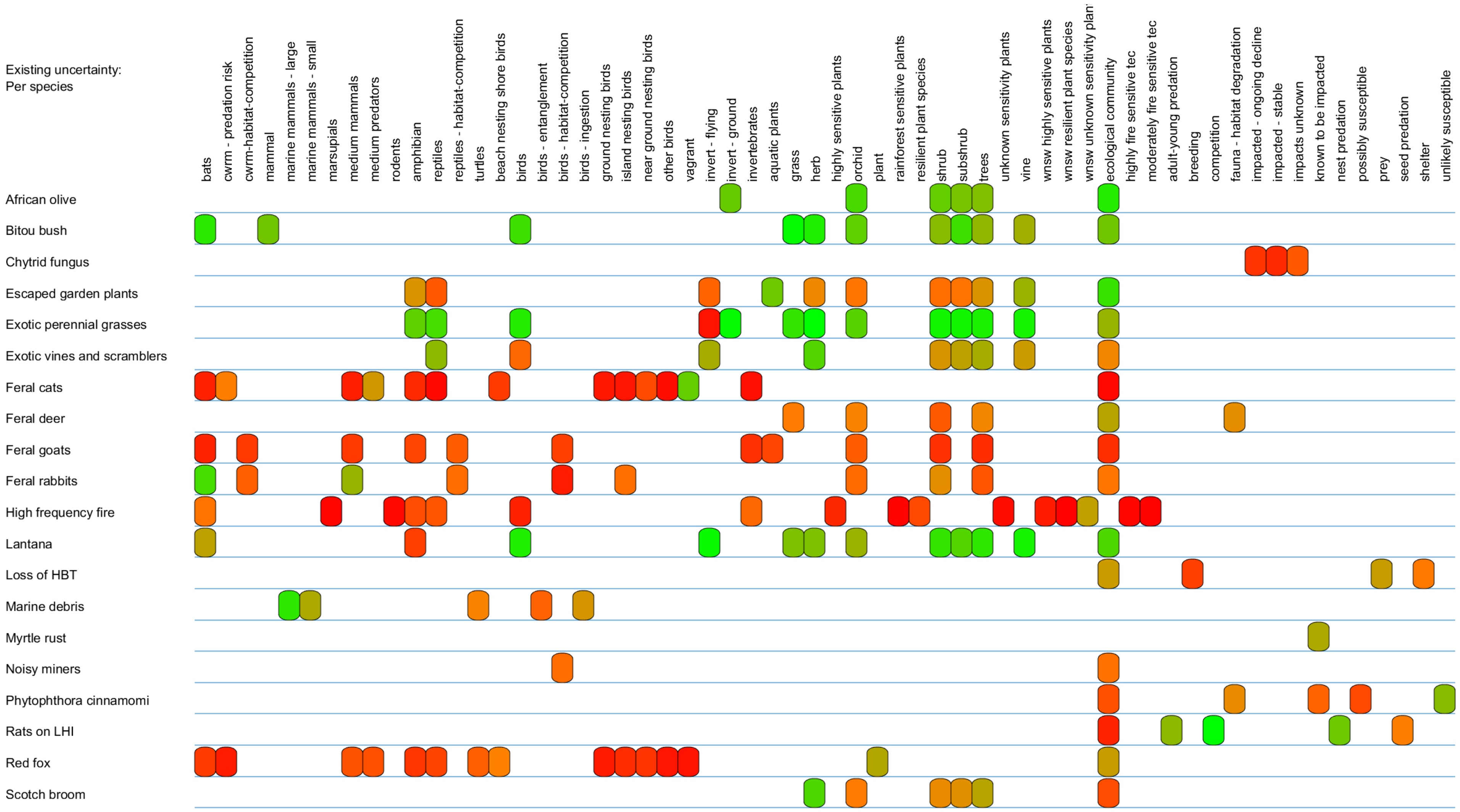

Supplementary Figure 1: Per-species expected gain in persistence under current uncertainty for each species group represented as percentile ranks. KTP-species group pairs with high expected gain in persistence are coloured green and are good options for immediate management; KTP-species group pairs with low expected gain in persistence are coloured red.

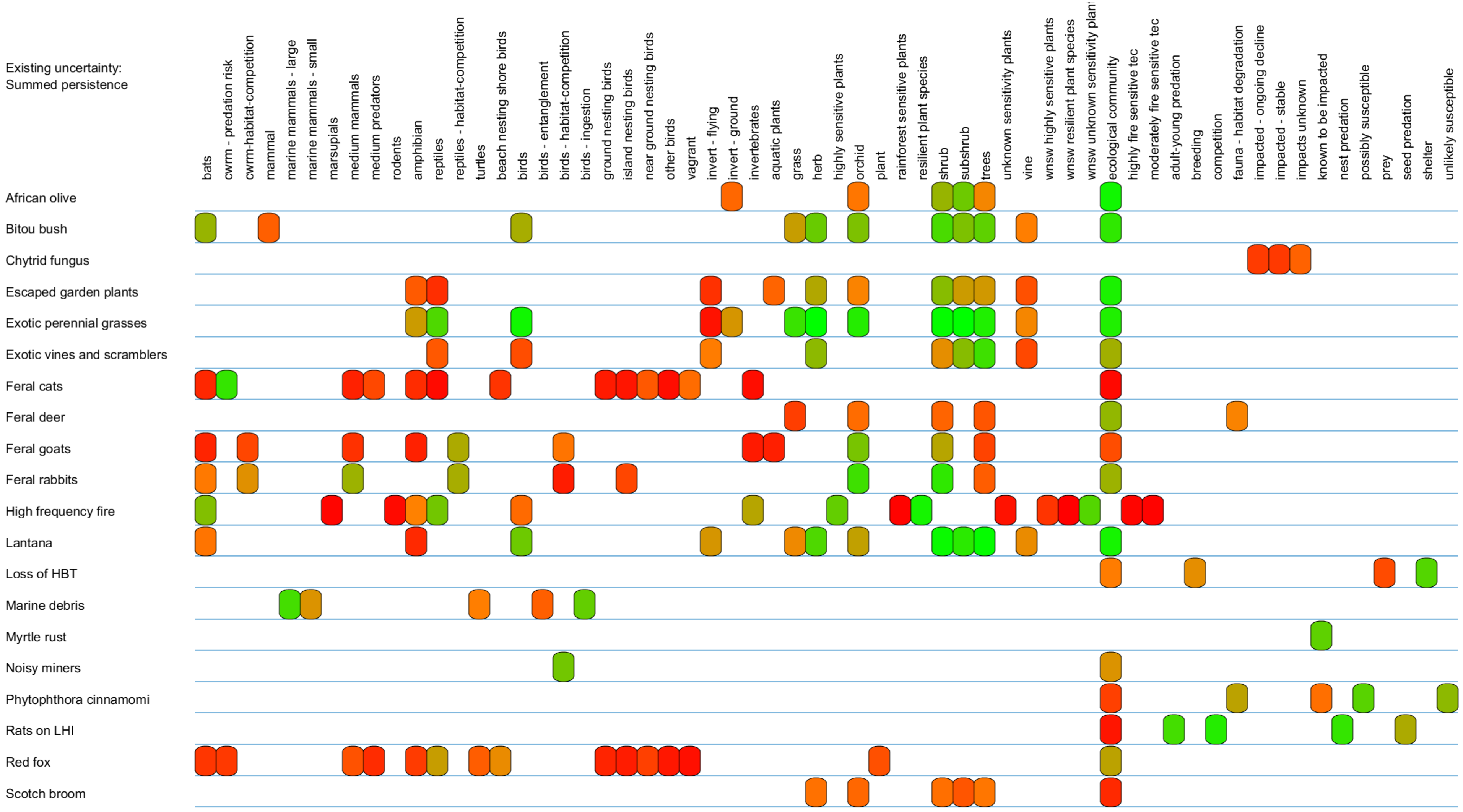

Supplementary Figure 2: Total expected gain in persistence summed over all species, under current uncertainty for each species group represented as percentile ranks. KTP-species group pairs with high expected gain in persistence are coloured green and are good options for immediate management; KTP-species group pairs with low expected gain in persistence are coloured red.

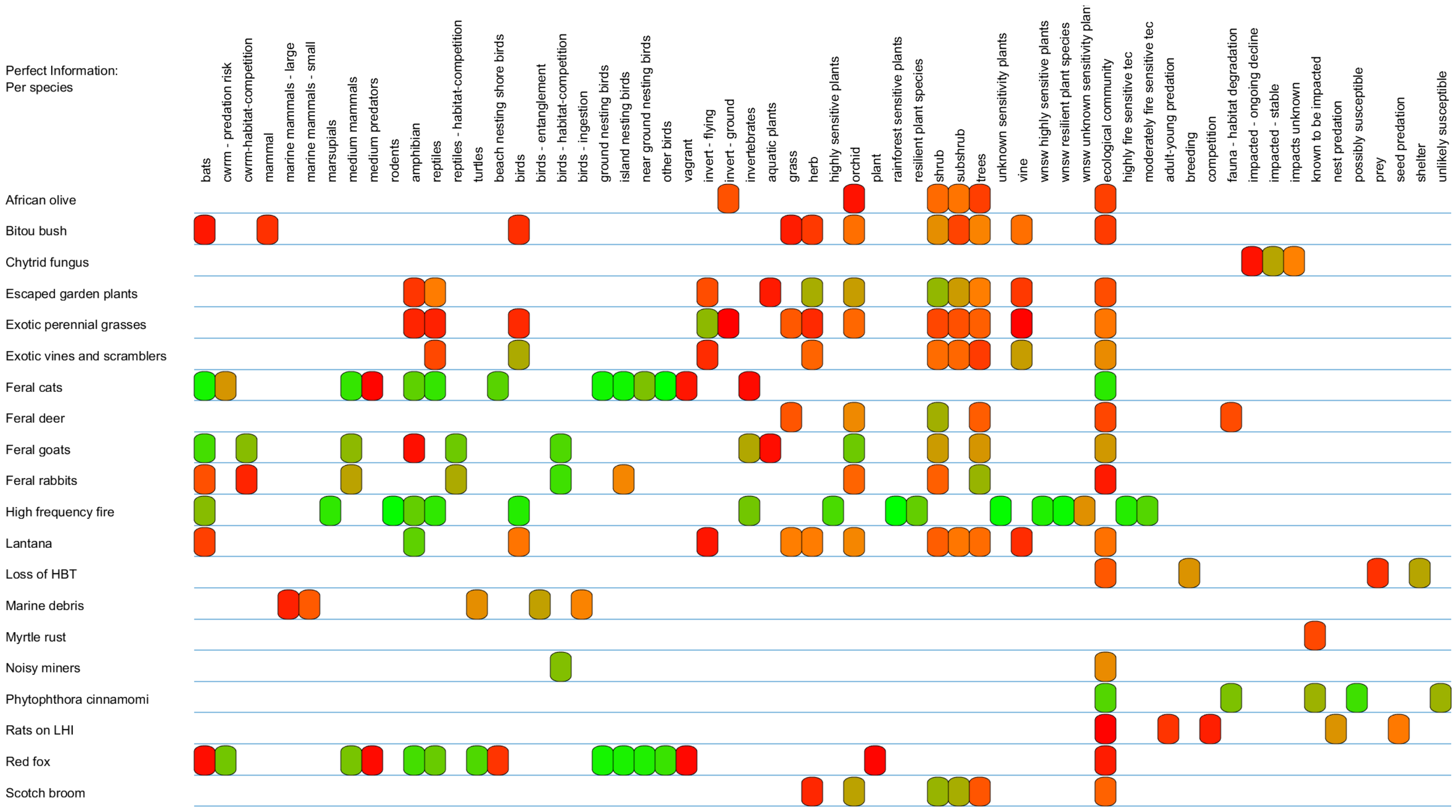

Supplementary Figure 3: Per-species expected EVPI for each species group represented as percentile ranks. KTP-species group pairs with high expected gain in persistence are coloured green and are good options for research to reduce uncertainty about management effectiveness; KTP-species group pairs with low expected EVPI are coloured red.

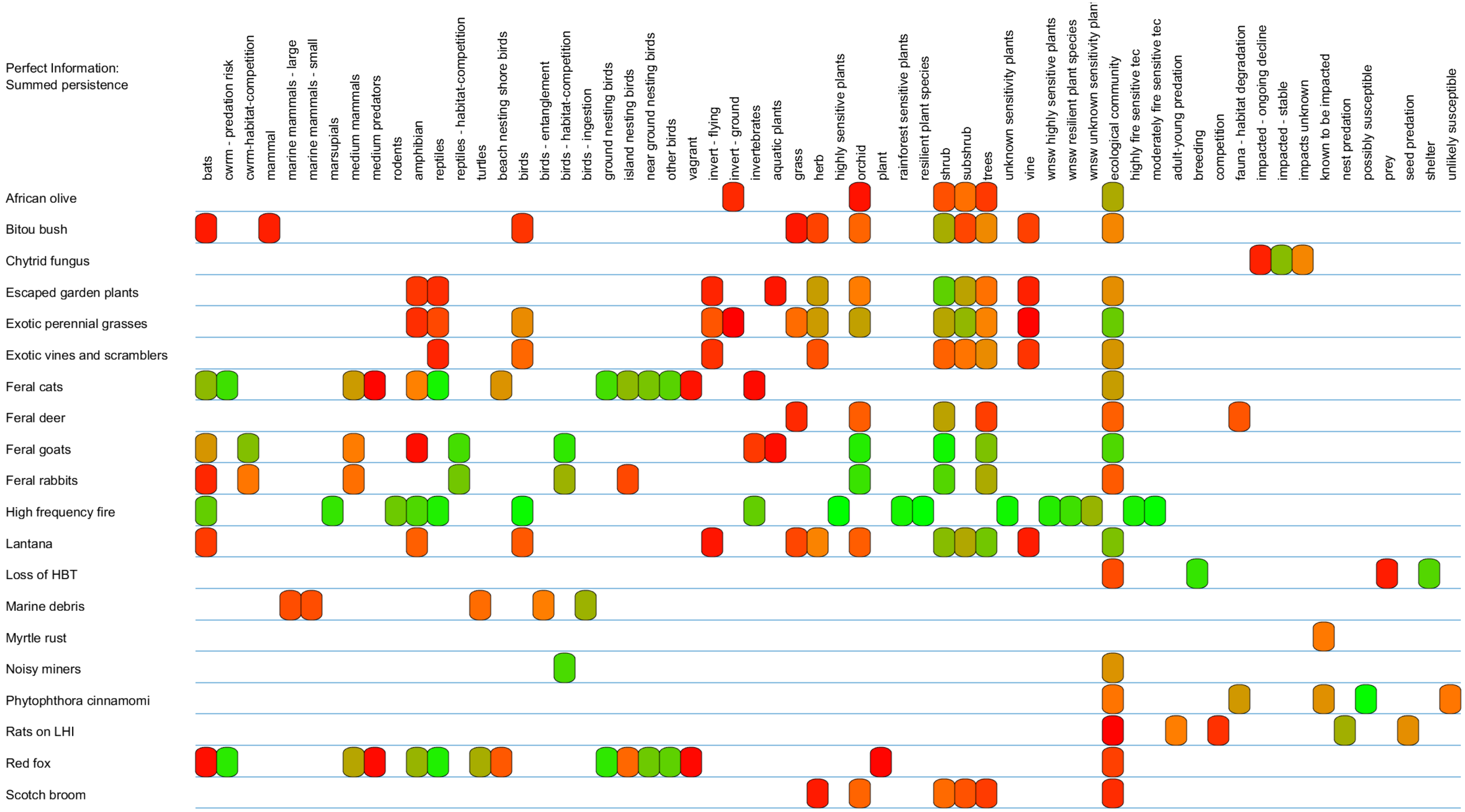

Supplementary Figure 4: Total expected EVPI for each species group, summed over all species, represented as percentile ranks. KTP-species group pairs with high expected gain in persistence are coloured green and are good options for research to reduce uncertainty about management effectiveness; KTP-species group pairs with low expected EVPI are coloured red.

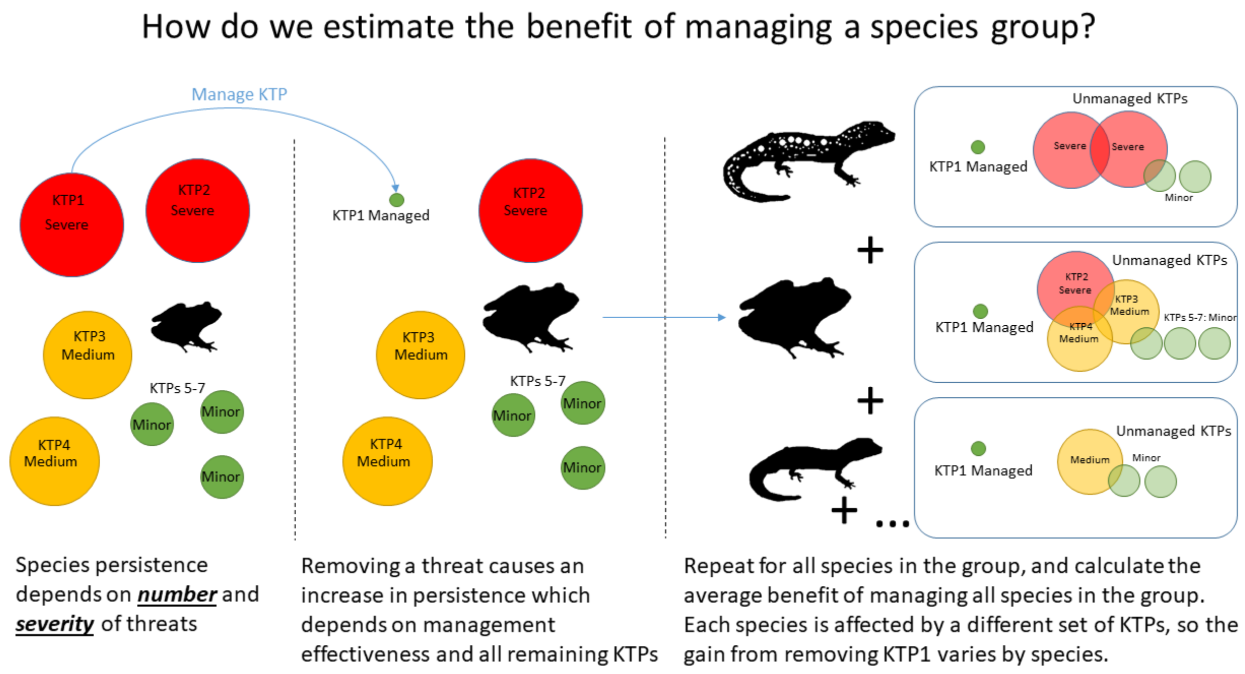

Supplementary Figure 5: Schematic of the process used to provide an initial estimate of the benefit (i.e. gain in persistence) of managing a species group. The benefit of managing a KTP is computed using a weighted average of the benefits of all species groups affected by the KTP, where weights are determined by the number of species in each species group. The initial estimate was provided to species experts as a guide only and could be overruled based on expert judgement.

Supplementary Tables

Supplementary Table 1: Number of listed species and ecological communities under State (New South Wales) and National (Australia) legislation.

Listing criterion	Number of affected species
**State listing**	
Critically Endangered	84
Endangered	413
Vulnerable	374
Critically Endangered Ecological Community	17
Endangered Ecological Community	84
Vulnerable Ecological Community	4
**Total State Listed Species**	**972**
**National Listing**	
Critically Endangered	58
Endangered	177
Vulnerable	238
**Total Nationally Listed Species**	**473**

Supplementary Table 2: Number of experts who contributed elicited data for each KTP. A KTP type has been added to aid readers unfamiliar with Australian threatening processes.

KTP number	KTP	KTP type	Number of experts contributing responses	Number of affected species
1	Noisy miners	Pest animal (competition)	4	29
2	Chytrid fungus	Disease/pathogen	3	23
3	Loss of hollow-bearing trees	Habitat loss	3	50
4	Phytophthora cinnamomi	Disease/pathogen	3	92
5	Rats on Lord Howe Island	Pest animal (island)	4	29
6	Marine debris	Marine debris	4	26
7	Myrtle rust	Disease/pathogen	4	8
8	Feral goats	Pest animal (herbivore)	2	195
9	Feral rabbits	Pest animal (herbivore)	1	116
10	Feral pigs*	Pest animal	1	132
11	Red fox	Pest animal (predator)	3	130
12	Feral cats	Pest animal (predator)	4	108
13	Feral deer	Pest animal (herbivore)	5	20
14	Exotic perennial grasses	Invasive plant	3	116
15	Exotic vines and scramblers	Invasive plant	3	40
16	Bitou bush	Invasive plant	6	40

**17**	Lantana	Invasive plant	2	82
**18**	Escaped garden plants	Invasive plant	4	57
**19**	Scotch broom	Invasive plant	3	12
**20**	African olive	Invasive plant	3	25
**21**	High frequency fire	Fire	1	885

Supplementary Table 3: Glossary of terms used in the text

Term	Notation	Description
Species group (when aggregated across species groups, can also represent a KTP)	i	A group of species that respond similarly to a KTP. The responses of all species groups affected by a KTP can be aggregated to the KTP level to obtain an average response for the KTP.
Management effectiveness	θ_i	A measure of the extent to which best-practice threat management can reduce the severity or extent of KTP i. Measured on a 0-1 scale, where 0 represents no effect of management and 1 represents complete removal of the threat. Obtained from expert elicitation.
Persistence without management	b_{0i}	Estimated probability of persistence in the absence of management action. This is the average per-species persistence of a species from either species group or KTP i, depending on the level of aggregation. Obtained from expert elicitation.
Persistence with management (or species response to management)	b_i	Estimated probability of persistence as a result of best-practice management action. This is the average per-species persistence of a species from either species group or KTP i, depending on the level of aggregation. Obtained from expert elicitation.
Benefit (or utility)	$b_i - b_{0i}$	Gain in the probability of persistence of species from group i (i represents either a species group or a KTP) as a result of management.
Expected value of management under current uncertainty	$EV_{uncertainty}$	Expected per-species gain in persistence if we were to act with current uncertainty. Can be computed at either the species group or KTP level of aggregation.
Expected value of management	$EV_{certainty}$	Expected per-species gain in persistence if we were to act after uncertainty about management effectiveness and species

with no uncertainty		response was removed. Can be computed at either the species group or KTP level of aggregation.
Expected value of perfect information (value of information)	$EVPI$	The difference between $EV_{certainty}$ and $EV_{uncertainty}$. Measures the improvement in benefit from removing uncertainty about management effectiveness and species response.
