# Supplementary material for: Quantifying the impact of uncertainty on threat management for biodiversity
Source: Nat Commun. 2019 Aug 8;10:3570. doi: 10.1038/s41467-019-11404-5 (PMC6687751; doi:10.1038/s41467-019-11404-5)
Supplement: Supplementary file 3 — Reporting Summary [file 41467_2019_11404_MOESM3_ESM.pdf]

nature research

Corresponding author(s): *Double-blind peer review submissions: write DBPR and your manuscript number here instead of author names.*

Last updated by author(s): *YYYY-MM-DD*

Reporting Summary

Nature Research wishes to improve the reproducibility of the work that we publish. This form provides structure for consistency and transparency in reporting. For further information on Nature Research policies, seeAuthors & Referees and theEditorial Policy Checklist .

Statistics

For all statistical analyses, confirm that the following items are present in the figure legend, table legend, main text, or Methods section.

n/a	Confirmed	
☒	☐	The exact sample size (n) for each experimental group/condition, given as a discrete number and unit of measurement
☒	☐	A statement on whether measurements were taken from distinct samples or whether the same sample was measured repeatedly
☒	☐	The statistical test(s) used AND whether they are one- or two-sided<br>*Only common tests should be described solely by name; describe more complex techniques in the Methods section.*
☒	☐	A description of all covariates tested
☒	☐	A description of any assumptions or corrections, such as tests of normality and adjustment for multiple comparisons
☒	☐	A full description of the statistical parameters including central tendency (e.g. means) or other basic estimates (e.g. regression coefficient) AND variation (e.g. standard deviation) or associated estimates of uncertainty (e.g. confidence intervals)
☒	☐	For null hypothesis testing, the test statistic (e.g. F, t, r) with confidence intervals, effect sizes, degrees of freedom and P value noted<br>*Give P values as exact values whenever suitable.*
☒	☐	For Bayesian analysis, information on the choice of priors and Markov chain Monte Carlo settings
☒	☐	For hierarchical and complex designs, identification of the appropriate level for tests and full reporting of outcomes
☒	☐	Estimates of effect sizes (e.g. Cohen's d, Pearson's r), indicating how they were calculated

Our web collection on statistics for biologists contains articles on many of the points above.

Software and code

Policy information about availability of computer code

Data collection	Data was collected manually-- no software was used for data collection.
Data analysis	Data was analysed with program R. Custom code used in the analysis is available in the figshare directory that includes the data (see data availability statement)

For manuscripts utilizing custom algorithms or software that are central to the research but not yet described in published literature, software must be made available to editors/reviewers. We strongly encourage code deposition in a community repository (e.g. GitHub). See the Nature Research guidelines for submitting code & software for further information.

Data

Policy information about availability of data

All manuscripts must include a data availability statement. This statement should provide the following information, where applicable:

- Accession codes, unique identifiers, or web links for publicly available datasets
- A list of figures that have associated raw data
- A description of any restrictions on data availability

All data for this manuscript, including anonymised expert inputs and R scripts, is available from: 10.6084/m9.figshare.7623665. The source data underlying Figures 1-2, 4-7 and the Supplementary Figures are provided as a Source Data file.

Field-specific reporting

Please select the one below that is the best fit for your research. If you are not sure, read the appropriate sections before making your selection.

Behavioural & social sciences study design

All studies must disclose on these points even when the disclosure is negative.

Study description	Our study develops a new technique to evaluate the value of information of resolving uncertainty about key threatening processes in the State of New South Wales, Australia. The study used expert elicited information to quantify the expected species persistence with and without threats acting on the population (and the associated uncertainty) as the basis for determining the value of information.
Research sample	Experts were ecologists or threatened species managers. 261 experts were invited to contribute by email, of which 66 provided estimates (summary of experts who contributed are included in Table 3). The study sample was chosen based on recommendations of the Office of Environment and Heritage, who are the agency responsible for managing threats and threatened species in the study area. The sample collected was likely to be representative as there are relatively few experts on specific threatened species; we consulted with the relevant agency to ensure that we gained information from the correct expertise.
Sampling strategy	We used a snowballing technique to add experts to the study. As above, there is no clear statistically significant number of experts for threatened species management, as the number of experts for any given species or threat tends to be quite low due to the specialist nature of threatened species management. The best way to determine expertise is via word of mouth, as experts tend to know other experts in this field. For this reason we used snowballing, relying on experts to tell us who was essential for the study credibility.
Data collection	Data was collected via email using an Excel template (included in the figshare data repository). Participants and their responses were kept anonymous from each other, although the researchers knew the identities of the researchers to enable us to follow up. We elicited data individually, then shared the results with all experts to allow experts to change their estimates after seeing the group's results. The research team contacted some individuals to confirm some estimates that had logical inconsistencies (e.g. best guesses lower than worst guesses), to ensure a minimum standard of data integrity.
Timing	Data was collected from 16/4/2018- 30/5/2018
Data exclusions	Data on feral pigs was excluded because we could not get an expert to provide data on the species responses to feral pig control (see manuscript "data collection" section). All other data was included.
Non-participation	261 experts were invited to contribute by email, of which 66 provided estimates. Those who dropped out either gave no response or cited a lack of time to be involved in the elicitation process. We had no objectors for other reasons.
Randomization	Not applicable to our study as we had a low number of participants and we did not recruit at random (we sought particular expertise rather than a standard sample of a population).

Reporting for specific materials, systems and methods

We require information from authors about some types of materials, experimental systems and methods used in many studies. Here, indicate whether each material, system or method listed is relevant to your study. If you are not sure if a list item applies to your research, read the appropriate section before selecting a response.

Materials & experimental systems

n/a	Involved in the study	
☒	☐	Antibodies
☒	☐	Eukaryotic cell lines
☒	☐	Palaeontology
☒	☐	Animals and other organisms
☐	☒	Human research participants
☒	☐	Clinical data

Methods

n/a	Involved in the study	
☒	☐	ChIP-seq
☒	☐	Flow cytometry
☒	☐	MRI-based neuroimaging

Human research participants

Policy information about studies involving human research participants

Population characteristics	See above
Recruitment	Participants were recruited using a snowball approach, as the best way to identify threatened species expertise is generally to ask known experts to nominate other recognised experts. All participants were either government scientists, ecological consultants or academics, so all had relevant credentials for the questions being asked.
Ethics oversight	CSIRO Social Science Human Research Ethics Committee (project ID: 006/18).

Note that full information on the approval of the study protocol must also be provided in the manuscript.
